# Minimally Invasive Surgical Techniques for Periodontal Regeneration: Preserving the Entire Papilla Without Dissection—A Narrative Review

**DOI:** 10.3390/jcm14124117

**Published:** 2025-06-10

**Authors:** Sylwia Jakubowska, Bartłomiej Górski

**Affiliations:** Department of Periodontology and Oral Mucosa Diseases, Medical University of Warsaw, Binieckiego 6 St., 02-097 Warsaw, Poland; bgorski@wum.edu.pl

**Keywords:** periodontal regeneration, intrabony defects, entire papilla preservation, interdental papilla, periodontics, soft tissue surgery

## Abstract

**Background:** The aim of the present narrative review is to synthesize the available scientific evidence on the minimally invasive surgical techniques for periodontal regeneration preserving the entire papilla without dissection. Surgical treatment of intrabony defects may result in compromising the integrity of the interdental tissues and subsequent papilla loss. Therefore, it is indicated to investigate the approaches avoiding papillary incision over the osseous defect, thus optimizing wound healing conditions. **Methods:** Authors performed a search of literature via electronic databases such as PubMed, Web of Science, Cochrane, and Scopus, and extended by manual searching with a stop date of February 2025. Based on inclusion criteria only randomized clinical trials (RCT), cohort studies, case–control studies, and case series were included, and 106 records were initially identified. Various aspects of described novel approaches preserving the entire papilla were finally discussed. **Results:** A total of 12 studies were evaluated. There is a significant lack of randomized controlled clinical trials on minimally invasive techniques without incision in the papilla. However, numerous modifications of existing techniques have emerged, mainly in the form of case series and case reports with short-term data. Among them, some authors stated that the entire papilla preservation approaches may facilitate early soft tissue healing, reduce papilla trauma and the risk of gingival recession, minimize procedure time, improve flap stability, and alleviate discomfort and side effects, while others reported similar outcomes to conventional approaches and emphasize the need for further comparative clinical trials. **Conclusions:** Preserving papilla integrity and the soft tissue profile is essential for minimizing complications, especially in the esthetic zone. Within the limitations of this narrative review, presented findings emphasize the effectiveness of entire papilla preservation techniques in preventing post-surgery tissue loss compared to conventional incisions and flaps. Randomized controlled trials with longer follow-up periods and larger sample sizes are necessary to validate the efficacy of these approaches in comparison to established papilla preservation techniques.

## 1. Introduction

Periodontitis is a persistent inflammatory condition of multifactorial origin, characterized by the gradual degradation of the periodontium. The associated alveolar bone loss may manifest in distinct morphological patterns—horizontal, angular, or vertical—frequently leading to the development of intrabony periodontal defects. These osseous defects are considered critical indicators of disease severity and progression, and they commonly necessitate regenerative periodontal interventions to restore lost tissue architecture and function [1,2,3]. After initial periodontal therapy, the persistence of pathological pockets with intrabony bone loss patterns is considered a significant risk factor for continued disease progression. These sites frequently require further surgical management to achieve favorable clinical results [2,3,4,5,6,7]. Extensive scientific evidence supports the effectiveness of periodontal reconstructive surgery in managing teeth with deep periodontal pockets and pronounced clinical attachment loss, contributing to sustained functional stability [8,9]. Although contemporary minimally invasive techniques for the treatment of intrabony defects have shown substantial success in reducing probing depths and improving clinical attachment levels, multiple studies have noted a consistent occurrence of marginal soft tissue contraction following surgery. Furthermore, despite overall favorable clinical outcomes, complete resolution of the intrabony defect is often not achieved [10,11,12]. In the majority of conventional minimally invasive periodontal regenerative techniques, the defect is accessed via incisions made in the marginal gingiva. However, this approach may present notable challenges, especially in cases where the incision is positioned at the base of the interdental papilla directly above the defect. The challenging anatomical features of the interdental area, combined with the specific morphology of intrabony defects, may predispose these sites to early wound dehiscence. Such complications heighten the risk of surgical site exposure, creating favorable conditions for bacterial contamination and the development of an inflammatory infiltrate, which are predisposing factors that may significantly compromise the success of the regenerative procedure [12,13]. Conventional marginal surgical techniques, characterized by sulcular incisions and the elevation of papillary and marginal soft tissues, are linked to an elevated risk of both horizontal and vertical tissue collapse. Functional forces and micromovements generated during mastication and routine oral hygiene can jeopardize clot stability at the root surface, potentially undermining the regenerative outcome. Additionally, the inherent difficulty in securing stable adaptation of marginal tissues to the root surface may contribute to esthetic concerns, such as postoperative soft tissue recession and irregular gingival contours [12,14]. Gingival recession (REC) often increases after surgical treatment of intraosseous defects, which is an undesirable outcome commonly linked to the selected flap design. Since gingival recession significantly affects oral health-related quality of life, it is highly recommended to adopt simplified surgical methods that reduce postoperative REC while maximizing clinical results [15]. Key components of contemporary minimally invasive surgical approaches include the utilization of magnification for enhanced visualization, limited flap extension and reflection to reduce tissue trauma, and conservation of the papilla and surrounding supracrestal tissues, supported by the application of scrupulous suturing. To minimize complications and enhance the outcome of PPT [16], MIST [17], M-MIST [18], and SFA [19], several innovative minimally invasive surgical procedures have been developed over the last years to preserve the entire papilla without dissection. This review summarizes records on Entire Papilla Preservation Technique (EPPT), Non-Incised Papillae Surgical Approach (NIPSA), the apically incised coronally advanced surgical technique (AICAST), Vestibular Incision Subperiosteal Tunnel Access (VISTA), and periodontal endoscopy-aided non-incisional regeneration technique (NIT), which are all periodontal regeneration techniques in the treatment of intrabony defects that avoid the traumatic incisions in at the papilla base. The principles are to maintain the integrity of the vascular supply, support optimal healing and reduce the risk of tissue necrosis. These approaches minimize surgical trauma, lower the likelihood of gingival recession or scarring, and preserve the natural gingival contour—key factors for achieving favorable esthetic outcomes. Additionally, they enhance soft tissue stability around grafts or biomaterials and contribute to improved patient comfort and faster recovery, making them highly valuable in minimally invasive periodontal surgery. Preventing complications in the esthetic region relies heavily on protecting the shape and integrity of the soft tissues between the teeth [20]. Therefore, it is indicated to investigate the approaches avoiding papillary incision over the osseous defect, thus optimizing wound healing conditions and advancing further than traditional PPT and MIST to reduce trauma. The aim of the present narrative review is to synthesize the available scientific evidence on the novel surgical techniques for periodontal regeneration preserving the entire papilla without dissection.

## 2. Materials and Methods

Search strategy and data extraction: an electronic search of the literature was conducted in February 2024. Four databases—PubMed/Medline, Web of Science, Cochrane, and Scopus—were screened for relevant articles. Prior to the screening process, the first 50 titles and abstracts retrieved by the electronic literature search were used for calibration of the two reviewers (S.J and B.G) with an independent supervising researcher (B.G). Agreement between reviewers was assessed using the kappa statistic. Consequently, titles and abstracts were independently screened by two reviewers (S.J and B.G). Authors obtained full texts of studies that potentially met the inclusion criteria and evaluated them for possible inclusion. Disagreement between the reviewers was discussed with an independent supervising researcher.

The first reviewer managed the article selection process by importing all records from the databases into Mendeley Reference Manager, where duplicates were removed and the articles were screened. The search strategy was adapted to each specific database and utilized Boolean operators to refine the results. The search was conducted using the following strategy: {(intervention) AND (outcome). (Intervention: [MeSH Terms] periodontal regeneration OR intrabony defects OR gingival recession OR clinical attachment level OR probing depth) AND (outcomes: [MeSH Terms] papilla preservation techniques OR minimally invasive surgery OR entire papilla preservation technique OR non-incised papillae surgical approach OR Soft tissue surgery)}.

The eligibility criteria for this narrative review were structured according to the PICOS framework: P (Population): studies on humans diagnosed with (a) periodontitis, (b) with the evidence of at least one deep intrabony defect, (c) in good general health, (d) with full-mouth plaque score (FMPS) and full-mouth bleeding score ≤ 20%.

I (Intervention): surgical techniques preserving the entire papilla.

C (Comparison): (a) papilla preservation techniques (PPTs), (b) MIST, (c) M-MIST.

O (outcome measures): average change in PPD, CAL, REC, periodontal tissue stability.

S (Study design): randomized clinical trials (RCT), case series, case–control studies, cohort studies. Only articles published in English were considered eligible.

The following were excluded: abstract-only publications, both narrative and systematic reviews, studies involving antimicrobial interventions, and animal or in vitro studies.

Data from the selected studies were extracted, organized into evidence tables, and visualized through figures such as flowcharts. The general findings were then synthesized in a narrative manner.

The risk of bias of included randomized clinical trials was assessed with The Cochrane Risk of Bias 2 tool for randomized trials (RoB2). The NIH Quality Assessment Tool for Case Series Studies was used to evaluate remaining 9 case series.

## 3. Results

**Figure 1:**
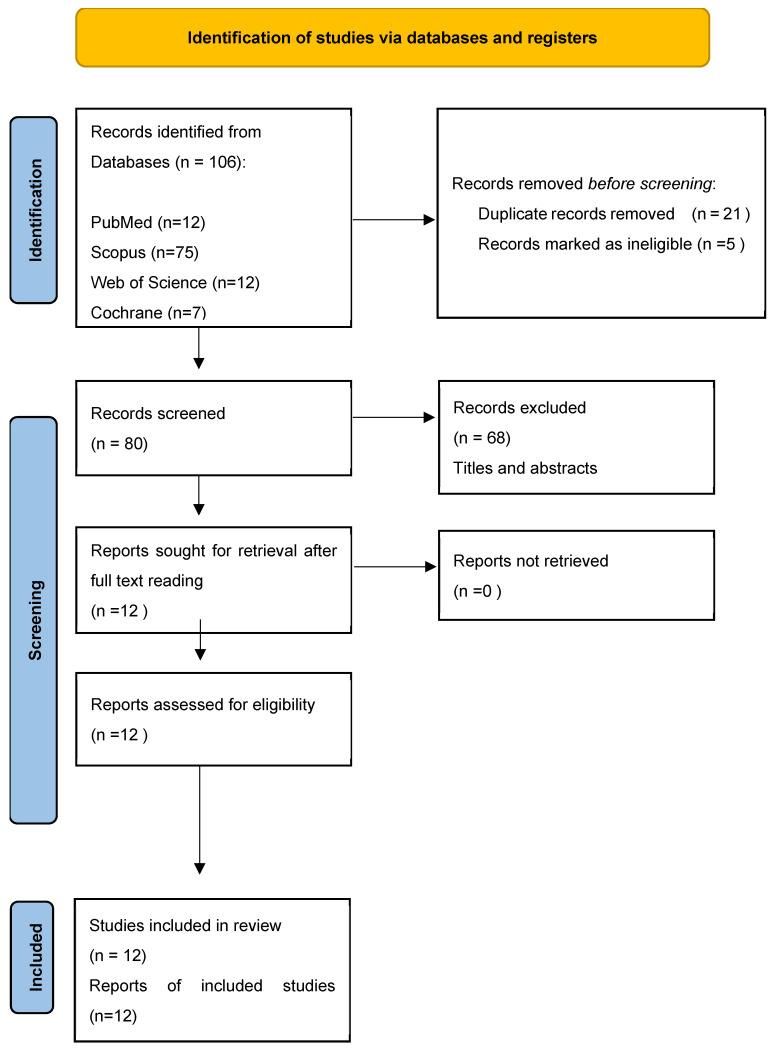
Flow chart of the selection process.

**Figure 2:**
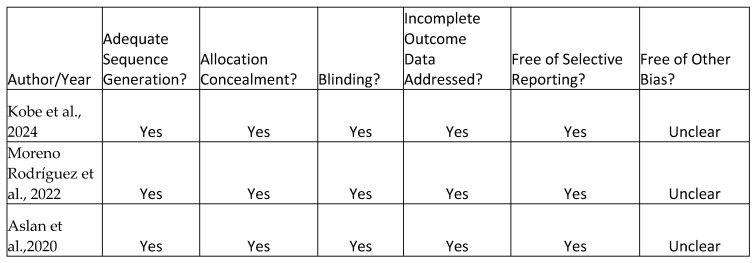
Summary of risk of bias of three included RCTs (ROB 2) [22,25,28].

**Figure 3:**
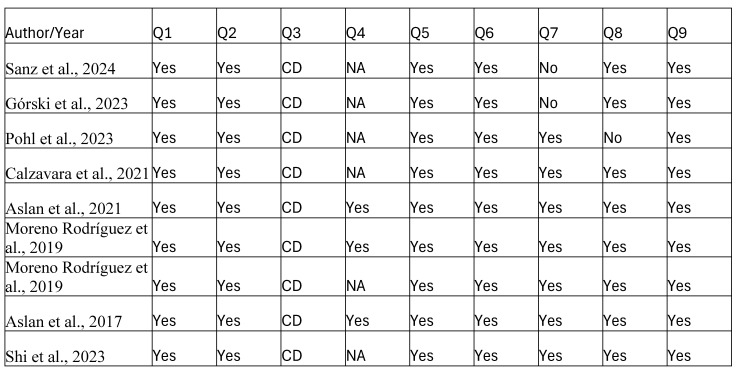
Summary of The NIH Quality Assessment Tool for Case Series Studies [21,23,24,26,27,29,30,31,32].

**Figure 4:**
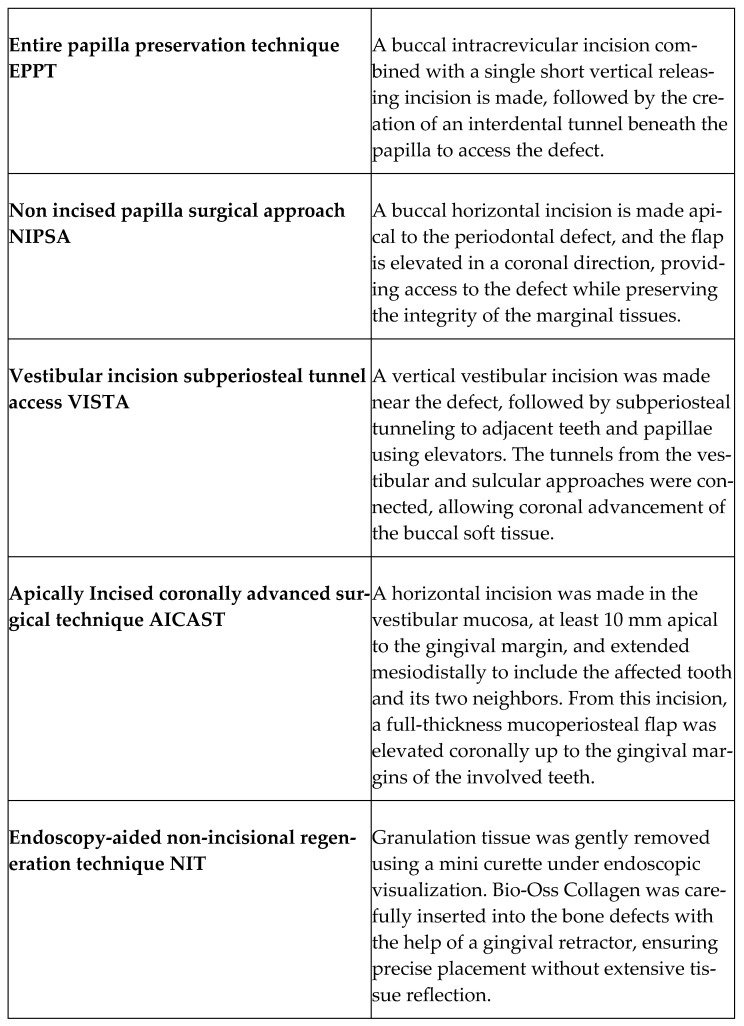
Short summary of included approaches.

A total of 106 articles were retrieved from the database searches [Figure 1]. Duplicates and studies that did not meet the predefined search criteria were subsequently eliminated. After abstracts revision, 68 studies were discarded. Following full-text evaluation, 12 studies focusing on the implementation and effectiveness of the novel surgical techniques for periodontal regeneration preserving the entire papilla without dissection were evaluated [21,22,23,24,25,26,27,28,29,30,31,32]. The main objectives of the chosen studies and summary of reported results are depicted in Table 1 (clinical relevance) and Table 2 (clinical outcomes). Included reports of non-incised entire papilla preservation techniques applications and their clinical relevance were finally discussed. Three RCTs [22,25,28] demonstrated an unclear risk of bias. Detailed risk of bias assessment is demonstrated in Figure 2. Six case series present good quality ratings [26,27,29,30,31,32], and three series present fair quality ratings [21,23,24]. A detailed assessment is depicted in Figure 3. A short summary of included approaches is depicted in Figure 4.

It is important to note that this review included different biomaterials, which were primarily used in two- and three-walled intrabony defects treated with various non-incision entire papilla preservation techniques.

Although levels of evidence are important in clinical and research decision-making, it is also essential to carefully evaluate the strengths and weaknesses of each individual study. In this review, only three randomized controlled trials and nine case series were included. Case series are limited by small sample sizes and the absence of control groups, which increases the risk of bias. Therefore, conclusions drawn from such studies should be interpreted with caution.

The clinical effectiveness of periodontal regeneration procedures may be reduced in high-risk populations, such as heavy smokers or individuals with inadequate oral hygiene. It is important to note that the majority of the studies included in this review involved only non-smokers or participants who had quit smoking at least one year prior to enrollment. Only RCT by Kobe et al. [22] and prospective case series by Calzavara et al. [26] allowed for inclusion patients smoking less than 10 cigarettes per day. They constituted 4 out of 20 patients in the Kobe et al. [22] trial and an unknown number in the Calzavara et al. [26] series.

Only one prospective case series by Sanz et al. [21] did not report the additional use of bone substitute materials or bone grafts in the surgical procedure. The EMD (Emdogain^®^; Straumann, Basel, Switzerland) was not reported in this series either. Moreno-Roudrigez et al. [25,30], Calzavara et al. [26], Shi et al. [32], and Aslan et al. [27,28,31] used deproteinized bovine-derived bone substitute (BS, Cerabone, Botiss Biomaterials GmbH, Berlin, Germany or Bio-Oss/Bio-Oss Collagen, Geistlich Biomaterials, Zurich, Switzerland), while Kobe et al. [22] reported that in the experimental group, a prehydrated collagen-containing corticocellular heterologous bone gel (OsteoBiol^®^ Gel, Tecnoss, Torino, Italy) was applied, and in the control group patient’s blood-soaked collagenous corticocellular xenogeneic bone graft (OsteoBiol^®^ Gen-Os, Tecnoss, Torino, Italy) was utilized. Meanwhile, Górski et al. [23] applied frozen, radiation-sterilized, allogeneic bone granules consisting of cortical and cancellous bone. Pohl et al. [24] were the first to use corticocancellous tuberosity bone (CCTB) for periodontal regeneration.

While the beneficial effects of EMD and its capacity to enhance regeneration have been widely recorded, two studies did not mention the additional use of amelogenins [21,22].

There was notable heterogeneity in the follow-up periods among the included studies. Two studies reported outcomes at 6 months [21,23], seven studies had a 12-month follow-up [22,25,27,28,30,31,32], and only two studies extended beyond 12 months [24,26]. Of these, one study reported long-term results for two cases with a follow-up of 5 years, highlighting the limited availability of long-term data in this area.

According to Aimetti et al. [6], two criteria for probing depth (PD)—≤3 mm and ≤4 mm—were assessed among studies. Each included study report a mean PD ≤ 4 mm at the follow-up for the treated sites, but not every study recorded the PD ≤ 3 mm after the observation period. In one RCT [25], both groups accomplished the residual PD < 5 mm (NIPSA EMD 2.50 ± 0.67 mm; NIPSA EMD + BS 2.67 ± 0.78 mm). The residual probing depth was 2 mm in 58.33% of cases treated with NIPSA EMD and in 50% of cases treated with NIPSA EMD + BS. A residual probing depth of 4 mm was observed in one case within the NIPSA EMD group and in two cases within the NIPSA EMD + BS group. In RTC using the EPPT [28] both groups showed a significant decrease in PD in the first years post-op. No significant inter-group differences were found (*p* = 0.866). In the EPP EMD + BS group, 33% of defects (n = 5) exhibited a residual probing depth of 2 mm, 53% (n = 8) had 3 mm, and 14% (n = 2) presented with a depth of 4 mm or more.

Among the EPP group, 20% of defects (n = 3) had a residual PD of 2 mm, 60% (n = 9) measured 3 mm, and another 20% (n = 3) had depths ≥ 4 mm. Some studies presented only mean PD difference values without percentage of reached pocket resolutions. In studies that did not use bone substitutes, the residual PD was 2.67 ± 1.03 mm (EPPT) and 2.59 ± 0.92 mm (AICAST) accordingly [21,26]. Meanwhile, cases series using the EPPT with BS and a collagen barrier membrane [27] reported residual PD 2.93 ± 0.59 mm after 1 year and a case series using the EPPT with BS recorded PD 2.67 ± 0.78 after 1 year [31].

Q1: Was the study question or objective clearly stated?, Q2: Was study population clearly and fully described, including case definition?, Q3: Were cases consecutive?, Q4: Were subjects comparable?, Q5: Was intervention clearly described?, Q6: Were outcome measures clearly defined, valid, reliable, and implemented consistently across all study participants?, Q7: Was length of follow-up adequate?, Q8: Were statistical methods well-described?, Q9: Were results well-described?, Good: Met 7–9 criteria, Fair: Met 4–6 criteria, Poor: Met 0–3 criteria. NA = not applicable, NIH = National Institutes of Health, NR = not reported.

In 2017, Aslan [31] published the first report on the tunnel-like surgical technique (EPPT) as 1 year follow-up of a prospective case series. The application of this technique supports the use of amelogenins and grafting materials. Three years later, an RCT was published by the same authors, Aslan et al. [28], where they combined the EPPT with biomaterials (EMD + BS). The outcomes were comparable to established methods, and it was documented that the use of the EPP, regardless of the use of adjunctive regenerative biomaterials, led to significant CAL gains and probing depth PD reductions, with minimal gingival recession. Moreover, authors concluded that the adjunctive use of regenerative biomaterials did not yield additional clinical benefits over EPP alone. In 2021, Aslan et al. [27] investigated another configuration of the EPPT in combination with GTR using native collagen membrane and bone grafting materials. According to the results, the technique may potentially provide effective protection for the biomaterial and optimal conditions for healing, even with the presence of a collagen barrier. The following authors went further to investigate the possibilities of the EPPT, and in 2023, Gorski et al. [23] proposed the modification to the original method design. In their modification, the buccal flap is extended mesiodistally in order to reduce flap tension and to enhance surgical access to a one-wall defect. Furthermore, the vertical releasing incision is lengthened as necessary to improve visualization. The findings of this study indicate that the modified EPPT, combined with EMD and allogenic bone grafting, may be considered as a potential treatment modality for intrabony defects. Follow-up of this case series demonstrated statistically significant and sustained clinical improvements over a 3-year period. The most recent prospective case series published by Sanz et al. in 2024 [21] evaluated the use of the EPPT without biomaterials for periodontal intrabony defect regeneration, assessing clinical and CBCT-based radiographic outcomes, as well as postoperative complications.

NIPSA was first proposed in 2018 (Moreno Rodriguez & Caffesse, 2018) [33]. One year later, the results of a prospective cohort study were published by the same authors [29]. It revealed that PD decrease was 5.53 ± 2.56 mm and CAL gain was 5.33 ± 2.47 mm. Moreover, the early wound healing index after one week was 1.5 ± 0.7, but recession increased by 0.20 ± 0.41 mm. The keratinized tissue width remained unchanged. In 2019, in the retrospective cohort study by Moreno Rodríguez et al. [30], the minimally invasive surgical technique (MIST) was compared with a non-incised papilla surgical approach (NIPSA). No significant differences between the two techniques were shown regarding PD reduction. However, authors report that after 1 year, the NIPSA group presented favorable, significant CAL gain (*p* < 0.05. It is suggested that a different soft tissue response (REC, TP, KT) is related to the approach used. The NIPSA design seems to minimize surgical trauma of tissues resulting in REC increase by only 0.2 ± 0.41 mm, while MIST resulted in the 0.73 ± 0.88 mm increase. Three years later, Moreno-Rodriguez et al. [25] presented an RCT in which they evaluate how non-incised papillae surgical approach (NIPSA) can be influenced by enamel matrix derivate (EMD) and bone substitutes (BS) in resolving combined non-contained periodontal defects with intrabony and supra-alveolar components. BoP was negative in all cases at the 1-year follow-up. Both groups demonstrated notable improvements, with substantial reductions in probing depth (NIPSA + EMD: 8.25 ± 2.70 mm; NIPSA + EMD + BS: 6.83 ± 0.81 mm) and gains in clinical attachment level (NIPSA + EMD: 8.33 ± 2.74 mm; NIPSA + EMD + BS: 7.08 ± 2.68 mm), all statistically significant (*p* < 0.001). However, no meaningful differences were detected in the inter-group comparison (*p* > 0.05). Soft tissue integrity was maintained in both groups, with no statistically significant differences observed between them (recession: NIPSA + EMD 0.25 ± 0.45 mm vs. NIPSA + EMD + BS 0.17 ± 0.58 mm, *p* > 0.05; keratinized tissue width: 0.00 ± 0.43 mm vs. 0.08 ± 0.67 mm, *p* > 0.05). Interestingly, although both groups exhibited improvements in papillary height, statistical significance was reached only in the NIPSA + EMD + BS group (0.45 ± 0.52 mm; *p* < 0.05), while the NIPSA + EMD group recorded a non-significant gain (0.33 ± 0.49 mm; *p* > 0.05).

Recently, in 2024, an RCT by Kobe et al. [22] was published, in which both papillae tunneling techniques (PTT), EPPT and NIPSA, were utilized in the treatment. Authors implemented prehydrated collagenated xenogenic bone gel in one group and collagenated cortico-cancellous heterologous bone mixture in the second group adjunctive to non-incised papilla preservation techniques. In this regard, incisors and canines were treated with NIPSA (n = 11) (Aslan et al., 2017) [31], whereas EPP was used with premolars or molars (n = 9). The authors noted that both surgical modalities ensured complete wound closure during the early healing phase and prevented biomaterial exposure in both the test and control groups.

In 2023, Pohl et al. [24] described a prospective case series using the VISTA approach with cortico-cancellous tuberosity bone grafting, debridement, EMD application, and CTG for periodontal regeneration. The authors used a single vertical incision just adjacent to the defect for gaining access and sulcular approach for root scaling. Visualization of the treated area was reported to be sufficient. The probing pocket depth improved from 8.2 ± 0.75 mm initially to 2.7 ± 0.52 mm at follow-up, clinical attachment level changed from 8.5 ± 0.83 mm initially to 2.7 ± 0.52 mm at follow-up, and gingival recession of 1 mm at two sites was corrected. The papillae remained stable across all sites, maintaining an average distance of 4.8 mm from the incisal edge to the papilla tip. Another innovative surgical design for preserving the entire papilla whilst avoiding dissection is the apically incised coronally advanced surgical technique (AICAST) proposed by Calzavara et al. in 2021 [26]. The case series presents the modification to surgical approach to reconstruct the interdental papilla by an apical incision in the lining mucosa described by Azzi et al. [34]. The modification facilitates the coronal advancement of both the gingival margin and the interdental papilla. In four out of nine cases where a buccal gingival recession was preoperatively present, a ctg was applied. CAL gains ≥ 6 mm were achieved in eight out of nine treated sites (88.9%), and PPD ≤ 3 mm was detected in all cases. Moreover, cases treated with an additional use of CTG resulted in a non-statistically significantly higher recession reduction and lower PPD reduction than the cases treated without the adjunctive use of ctg.

In 2023, a new non-incisional periodontal regeneration technique was proposed by Shi et al. [32]. In a retrospective cohort study, the authors analyzed the periodontal endoscopy-aided non-incisional regeneration technique (NIT) for the management of intrabony defects. In NIT, full access to the defect was achieved using periodontal endoscopy instead of flap elevation. Bone substitutes were then placed into the thoroughly debrided defect under endoscopic guidance. The NIT method was proposed as a further effort to reduce the invasiveness of periodontal regeneration surgery. The study aimed to explore the feasibility of periodontal endoscopy-aided NIT and to compare its effectiveness with periodontal endoscopy-aided SRP (PSRP). After 1 year, significant improvements in probing depth (PD), clinical attachment level gain (CAL), intrabony defect (IBD) depth, and gingival recession (GR) were demonstrated in both groups. Changes in these parameters between baseline and 1 year were statistically significant in both the NIT and PSRP groups (*p* < 0.001). Interestingly, CAL improved more in the NIT than the PSRP (*p* = 0.012). Regarding the changes in the GR no inter-group differences were noted (*p* = 0.232).

## 4. Discussion

This review was focused on the clinical outcomes of the novel surgical techniques for periodontal regeneration preserving the entire papilla without dissection. Maintaining papilla integrity and the soft tissue profile has tremendous value in periodontal regenerative surgery, especially in the esthetic area. The findings of this study are analyzed in comparison to the existing literature on the pre-established regenerative approaches, of which the core is to preserve the papilla and minimize the invasiveness of surgery. The recent EFP S3-Level Clinical Practice Guideline advocates for the use of specific flap designs that prioritize the preservation of interdental soft tissues, such as papilla preservation flaps [4,5]. In certain cases, minimizing flap elevation is also recommended to enhance wound stability and reduce patient morbidity [4]. Clinical outcomes of each included study showed a beneficial result in terms of average change in CAL, PD, and differences in REC during the follow-up. In a majority of studies, a positive change in CAL and PD exhibited a statistically significant value [21,23,27,29,30,31].

Conventional periodontal surgery frequently results in postoperative gingival recession, characterized by a concave contour at the coronal portion of the papilla. This morphology tends to promote plaque accumulation and persistent soft tissue inflammation. Additionally, the interdental papilla faces challenges in tissue regeneration due to its limited blood supply and the difficulty in achieving tension-free wound closure during the early healing phase. Research has demonstrated that guided tissue regeneration surgery (GTRS) is often associated with wound dehiscence and barrier membrane exposure, complications that compromise the stability and integration of bone graft materials, particularly in the interdental region. A recent systematic review and meta-analysis by Nibali et al. [35] concluded that guided tissue regeneration (GTR) offered additional benefits over open flap debridement (OFD), with gains of 1.15 mm in clinical attachment level (CAL-G) and 1.24 mm in probing pocket depth reduction (PPD-R) at 12 months. Furthermore, the incorporation of deproteinized bovine bone mineral (DBBM) further enhanced GTR outcomes, resulting in a CAL gain of 1.5 mm and a PPD reduction of 1.13 mm. Interestingly, the analysis also indicated that papillary preservation flaps led to improved clinical outcomes compared to conventional access flaps. This finding may suggest that, when performing regenerative procedures to treat intrabony defects, papillary preservation techniques should be considered the surgical approach of choice. [7]. With the development of new regenerative materials, such as enamel matrix derivatives, the use of barrier membranes is no longer essential for achieving successful outcomes. Papilla preservation flap techniques have progressed over time, beginning with traditional approaches and later advancing to the modified PPT (Checchi et al., 2009) [36], followed by the simplified PPT by Di Tullio et al. in 2013 [37]. In these techniques, the incision for the papilla preservation technique (PPT) is made at the papillary base. In spite of offering a reduction in papilla trauma compared to traditional methods, these methods still require mesio-distal dissection through the papilla. As a result, biomaterials placed beneath the incision line remain vulnerable to exposure. After one year, the SPPF with EMD showed significantly greater PD reduction (3.4 ± 0.7 mm), CAL gain (2.8 ± 0.8 mm), and less GR increase (0.6 ± 0.4 mm) compared to the non-EMD group (PD: 2.2 ± 0.8 mm; CAL: 1.0 ± 0.6 mm; GR: 1.2 ± 0.7 mm) (*p* < 0.001). The minimally invasive surgical technique (MIST), introduced by Cortellini et al. in combination with the use of an enamel matrix derivative (EMD), was developed to minimize both the mesio-distal extension and the corono-apical reflection of the flap. This approach aims to reduce surgical trauma while enhancing flap stability [17]. At 1-year follow-up, authors reported a significant clinical attachment level gain of 4.9 ± 1.7 mm (*p* < 0.0001) in comparison to the baseline and an increase in gingival recession of 0.4 ± 0.7 mm.

The single-flap approach (SFA) was introduced by Trombelli et al. in 2009 [19,38]. The SFA involves flap elevation on only one side (buccal or oral), preserving the opposite side. PD reduced from 9.0 ± 2.8 mm to 3.8 ± 1.5 mm, while GR increased slightly from 2.2 ± 1.9 mm to 2.6 ± 1.3 mm post-surgery. The Single Flap Approach (SFA) has shown promising results in minimizing postoperative gingival recession (REC), with six out of seven prospective clinical studies reporting mean REC changes of less than 1 mm at six months following surgery. Moreover, compared to papilla preservation techniques, SFA was associated with a lower occurrence of postoperative REC in both conservative and regenerative treatments of intraosseous defects [39].

While previous surgical techniques have significantly minimized trauma to the interdental papilla, they still fail to fully preserve its vascular supply. In cases involving isolated and deep intrabony defects, the use of suboptimal flap designs may markedly compromise the efficacy of regenerative treatment.

In the EPP technique [31], the initial surgical step involves making a vertical incision at the buccal line angle of the affected tooth, located distal to the osseous defect, along with an intrasulcular incision directed toward the defect. This design allows entry to the bony lesion through a tunneled approach. The papilla is elevated as a full-thickness flap, while preserving its natural, uninterrupted blood supply. This ensures optimal vascular perfusion, which helps reduce the risk of wound exposure. The authors reported that all sites healed without complications in the early stage, and at 1-year follow-up, all sites maintained a primary wound closure. EPP without the addition of biomaterials resulted in CAL gain of 5.83 ± 1.12 mm, PD reduction of 6.2 ± 1.33 mm, and gingival margin reduction of 0.36 ± 0.54 mm. According to Górski et al., modification to the EPPT maintained significant improvements from baseline at the 3-years follow-up. The authors reported significant reductions in PPD (7.03 ± 1.61 to 3.33 ± 0.89 mm, *p* < 0.0001), CAL improvement (to 3.08 ± 1.16 mm, *p* < 0.001), and DD decrease (4.59 ± 1.24 to 0.38 ± 0.31 mm, *p* < 0.001), while changes in gingival recession and keratinized tissue were not statistically significant [40].

The Non-Incised Papillae Surgical Approach (NIPSA) is founded on the principle of performing a single horizontal incision on the buccal mucosa, positioned as apically as possible relative to both the periodontal defect and the marginal soft tissues [29,30,33]. This technique entails the elevation of a coronally advanced mucoperiosteal flap, which allows apical access to the defect while maintaining the integrity of the marginal tissues. The preserved marginal tissue acts as a protective covering over the interproximal defect, supposedly helping to maintain soft tissue architecture and prevent papillary collapse. While the supraperiosteal gingival vessels neighboring the mucogingival junction are transected, the continuity of the non-incised gingival vasculature with the periodontal ligament is preserved, along with a strong lingual blood supply. This preservation of vascular integrity may provide an advantage over traditional extended flap techniques by ensuring enhanced perfusion to the surgical site [29,30].

The clinical outcomes reported in the case series by Calzavara et al. [26] utilizing the AICAST flap design are comparable to those reported in clinical trials using the MIST, M- MIST, SFA, and EPPT. However, the AICAST suggests additional suprabony clinical attachment gains in comparison to forementioned papilla preservation techniques. According to Calzavara et al. [26], the AICAST demonstrates positive performance in treating deep isolated intrabony lesions and the long-term maintenance of this outcomes. In study by Calzavara et al. [26], after the surgery, healing was 100% uneventful, and primary closure was achieved in all cases. At the last follow-up (5 years), PPD reduced by 6.05 ± 1.76 mm, and CAL gained 7.20 ± 2.13 mm (both *p* < 0.01), while the REC reduction of 1.15 ± 1.97 mm was not significant. The authors’ proposed modification enables the gingival margin and interdental papilla to be advanced coronally, creating an additional vertical space. When this space is filled with a scaffold, it has the potential to extend the attachment gain vertically, including the suprabony component of the defect. Furthermore, this coronal repositioning may correct existing gingival recessions and deficiencies in the interdental papillae, therefore enhancing the esthetic results. It has to be highlighted that this was the first publication of this technique, and the results have to be interpreted with caution. In order to minimize the post-operative gingival recession and patient discomfort, Shi et al. [32] performed a retrospective analysis after 1 year where 21 subjects were treated with non-incisional regeneration technique (NIT) and 21 subjects underwent periodontal endoscopy-aided scaling and root planning (PSRP). Based on the hypothesis that the stability of blood clot can improve without open flap, this study presents a promising approach for enhanced healing by creating an optimal periodontal microenviorment. Both groups showed significant improvements in PD, CAL, and IBD, along with an increase in gingival recession over the follow-up period. The NIT group demonstrated significantly greater CAL gains compared to PSRP (*p* = 0.012). In NIT-treated sites, 32.7% achieved ≥5 mm CAL gain, and 52.7% showed ≥4 mm PD reduction. The average CAL gain of 3.62 ± 2.70 mm in the NIT group aligns with outcomes reported in other studies using GTR or EMD-based regenerative therapies. The periodontal endoscopic system allows for direct visualization of subgingival biofilms, root surfaces, and calculus within periodontal pockets. Through real-time, magnified imaging, it enables clinicians to perform precise and targeted debridement of subgingival deposits, potentially making periodontal therapy less invasive. While traditionally used to improve the effectiveness of scaling and root planing (SRP) by allowing accurate removal of biofilms and calculus, its capacity to visually access the walls of intrabony defects also highlights its potential in regenerative procedures—specifically by facilitating defect debridement without the need for flap elevation [41]. The studies included in the presented review are characterized by keeping the flap as short as possible, with minimal exposure to the residual bone crest reaching a stable primary wound closure to seal the regenerated region and permitting an incident-free healing phase. Clinical outcomes are comparable to pre-established papilla preservation techniques in terms of clinical attachment gain and periodontal depth reduction. However, the majority of the presented studies report minimal post-operative gingival recession, maintenance, or even coronal advancement of the gingival margin, suggesting that the approaches may also enhance the esthetic outcomes. On the other hand, Rasperini et al. [42] suggest that the combination of conventional papilla preservation techniques with connective tissue grafts and coronal flap advancement can promote intrabony defect regeneration while simultaneously achieving interproximal root coverage and papilla reconstruction.

Several key factors must be taken into account when interpreting scientific data on techniques preserving entire papilla.

Establishing a threshold for pocket resolution is essential, as the chosen cut-off values influence how treatment success is defined. According to Aimetti et al. [6], two criteria for probing depth (PD)—≤3 mm and ≤4 mm—were considered in the results. A PD of ≤3 mm reflects the physiological depth of a healthy gingival sulcus, aligning with the ultimate goal of periodontal regeneration—to restore lost attachment. In contrast, a PD of ≤4 mm is often used to indicate periodontal stability in successfully treated patients, particularly when there are no sites with PD > 4 mm or PD = 4 mm with bleeding on probing [4,5]. Aimetti et al. [6] support the declining use of non-resorbable membranes in the last decade, likely due to their association with a high rate of complications and the requirement for a second surgical procedure for membrane removal. However, they also state that the probability of pocket resolution was higher for regenerative procedures than PPTs in meta-analysis for both thresholds of treatment outcomes, but there was a heterogeneity in surgical techniques used. They also state that greater weighted mean reductions in probing depth (PD) and gains in clinical attachment level (CAL) were observed in intrabony defects treated with enamel matrix derivative (EMD) combined with biomaterials or non-resorbable membranes, compared to other regenerative approaches. Additionally, papilla preservation techniques (PPTs) used alone were less effective than guided tissue regeneration (GTR) in achieving PD reduction and CAL gain, as shown in the pairwise meta-analyses. Therefore, authors of this review emphasize the need for randomized controlled trials comparing the use of EPPT without papilla dissection in conjunction with regenerative procedures to determine their superiority or inferiority.

The techniques have certain limitations and drawbacks, which the authors acknowledge and are aware of. The EPPT, NIPSA, and VISTA approaches would not be appropriate if the defect involved the lingual bone crest. The AICAST was reported specifically for only one or two-wall defects with a missing buccal bone on a single rooted teeth. VISTA was advised for use in the anterior region, whereas EPP was not preferred for this area due to the potential for visible scarring along the vertical incision line. EPPT was also not suggested for a narrow interproximal space, because of an increased risk of rupturing the fragile papilla [20]. The disadvantage of NIPSA is the risk of cutting-off the blood supply through a horizontal incision. Suturing in the EPP technique was the simplest, requiring only interrupted sutures, whereas NIPSA and VISTA necessitated more advanced methods such as horizontal mattress and sling sutures. In the NIT [32], case selection is a critical issue. The use of endoscopic instruments may be limited by narrow pocket openings, furcation morphology, or restricted mouth opening. In thin-scalloped biotypes, there is a risk of gingival stripping. Root anomalies like enamel pearls or palatal grooves can hinder NIT application.

A follow-up period of more than 12 months is preferred in periodontal regeneration studies because it allows for the evaluation of long-term stability, true tissue regeneration, and the detection of any delayed complications or relapse. It provides more reliable and clinically meaningful evidence compared to shorter-term follow-up. However, there is a heterogeneity among the included records and only two studies presented follow up period exceeding 12 months [24,26].

Most of the studies were prospective, retrospective and nonrandomized, and there was majority of small sample case series, insufficient to fully detail the procedures. One has to keep in mind that three RCTs and three case series presented an unclear or fair risk of bias, while six case series were assessed good quality, which could also influence the overall summary.

More robust evidence could be gained from randomized clinical trials and multicenter studies. There is a need for large, prospective, and well-designed research in this area. Lastly, the limitations inherent to the design of this narrative review, including a higher degree of bias, should be taken into account before drawing conclusions [43].

## 5. Conclusions

Within the limitations of this narrative review, it cannot be conclusively determined whether entire papilla preservation techniques that avoid papilla base dissection represent a viable or superior therapeutic approach for the regenerative treatment of intrabony defects. Clinical results of the analyzed studies seem to be comparable to those achieved with conventional papilla preservation techniques. Some findings also report the effectiveness in preventing post-surgery papilla loss, emphasizing the importance of selecting the appropriate technique based on the defect area and patient esthetics. Therefore, the authors indicate a possible area of interest and research for the future on innovative treatment modalities further reducing invasiveness of periodontal regenerative surgical procedures.

## Figures and Tables

**Table 1:** Main characteristics of the selected studies and summary of objectives and clinical relevance.

Author/Year	Title	Type of Article	Objectives	Clinical Relevance
Sanz et al., 2024 [21]	“Entire papilla preservation technique for treatment of periodontal intrabony defects: a series of cases.”	Prospective case series	To evaluate the use of EPPT without biomaterials for periodontal intrabony defect regeneration, assessing clinical and CBCT-based radiographic outcomes, as well as postoperative complications.	Study demonstrates that the EPPT improves key clinical parameters (PD reduction, CAL gain) without grafts. CBCT enabled precise defect assessment and confirmed post-treatment bone formation, supporting the EPPT as a regenerative, minimally invasive, and cost-effective approach.
Kobe et al., 2024 [22]	“Prehydrated collagenated cortico-cancellous heterologous bone gel and papillae tunneling for isolated intrabony defects: 12-month noninferiority trial.”	Randomized, controlled clinical trial	To determine how prehydrated collagenated xenogenic bone gel and a collagenated cortico-cancellous heterologous bone mixture together with EPP or NIPSA are effective in periodontal regenerative procedures.	Results indicate that prehydrated collagen cortico-cancellous bone gel combined with papilla-preserving procedures (EPP or NIPSA) produces similar reductions in PD and clinical attachment gain, with only minor gingival recession compared to conventional slowly resorbable solid particulate bone graft substitutes.
Górski et al., 2023 [23]	“Entire Papilla Preservation Technique with Enamel Matrix Proteins and Allogenic Bone Substitute for the Treatment of Isolated Intrabony Defects: A Prospective Case Series.”	Prospective case series	To evaluate clinical and radiographic outcomes of a modified EPPT with extended buccal flap elevation, combined with EMD and radiation-sterilized allogenic grafts for treating isolated intrabony defects.	Study suggests that the modified EPPT may benefit isolated intrabony defects. However, results are based on a single treatment concept without a control group and influenced by variables like SCTG. These results should be considered pilot data, and further trials are needed to evaluate each component’s role. Given similar results to established methods, justifying combination therapy remains challenging.
Pohl et al., 2023 [24]	“VISTA Approach in Conjunction with Enamel Matrix Derivative, Corticocancellous Bone, and Connective Tissue Graft for Periodontal Defect Surgery: A Case Series.”	Prospective case series	To describe the VISTA approach with CCTB (cortico-cancellous tuberosity bone) grafting, debridement, EMD application, and CTG for periodontal regeneration and soft tissue maintenance in the anterior region.	Results suggest that the VISTA approach with EMD, CCTB, and CTG is a promising technique for regenerating periodontal defects with intact lingual bone.
Moreno Rodríguez et al., 2022 [25]	“Apical approach in periodontal reconstructive surgery with enamel matrix derivate and enamel matrix derivate plus bone substitutes: a randomized, controlled clinical trial.”	Randomized controlled clinical trial	To assess whether the effectiveness of the non-incised papillae surgical approach (NIPSA) could be impacted by additional use of bone substitutes (BS) and enamel matrix derivative (EMD). Report describes deep, isolated, non-containing defects combining intrabony and supra-alveolar components.	Regardless of whether bone substitutes were used, the NIPSA technique demonstrated significantly improved clinical outcomes along with effective preservation of soft tissue integrity. The use of bone substitutes may promote interproximal soft tissue gain.
Calzavara et al., 2021 [26]	“The apically incised coronally advanced surgical technique (AICAST) for periodontal regeneration in isolated defects: a case series.”	Retrospective case series	To report the performance of AICAST (the apically incised coronally advanced surgical technique) in treating non-containing periodontal intrabony defects.	AICAST represents a recent treatment approach. Although study is based on a single treatment approach, preliminary outcomes present favorable clinical attachment gains and preservation of the soft tissues with eventual reduction in the associated gingival recessions.
Aslan et al., 2021 [27]	“Reconstructive surgical treatment of isolated deep intrabony defects with guided tissue regeneration using entire papilla preservation technique: A prospective case series.”	Prospective case series	To evaluate how effective is the entire papilla preservation (EPP) in combination with native collagen membrane and bone grafting materials in periodontal regeneration.	Presented technique, which avoids incision of the interdental papilla associated with the defect, appears promising for ensuring optimal biomaterial protection and healing conditions, even when a collagen barrier is used.
Aslan et al., 2020 [28]	“Clinical outcomes of the entire papilla preservation technique with and without biomaterials in the treatment of isolated intrabony defects: A randomized controlled clinical trial.”	Randomized controlled clinical trial	To evaluate and compare the clinical effectiveness of the entire papilla preservation technique (EPP), both as a standalone approach and in combination with enamel matrix derivatives and bovine-derived bone substitutes (EPP + EMD + BS), in the treatment of isolated interdental intrabony periodontal defects	The use of the EPP, both with and without adjunctive regenerative biomaterials, led to significant CAL gains and probing depth PD reductions, with minimal gingival recession. The adjunctive use of regenerative biomaterials did not yield additional clinical benefits over EPP alone
Moreno Rodríguez et al., 2019 [29]	“Supra-alveolar attachment gain in the treatment of combined intra-suprabony periodontal defects by non-incised papillae surgical approach.”	Prospective cohort study	To evaluate the clinical applicability of NIPSA in managing both intrabony and supra-alveolar components of periodontal defect.	Principal findings show that NIPSA improved clinical outcomes, including the supra-alveolar component, reducing soft tissue collapse risk. NIPSA may be a promising approach in periodontal reconstructive surgery, enhancing esthetic outcomes through optimal supra-alveolar tissue stability.
Moreno Rodríguez et al., 2019 [30]	“Periodontal reconstructive surgery of deep intraosseous defects using an apical approach. Non-incised papillae surgical approach (NIPSA): A retrospective cohort study.”	Retrospective cohort study	To compare a minimally invasive surgical technique (MIST) and a non-incised papilla surgical approach (NIPSA) in periodontal reconstructive surgery of deep intraosseous defects.	NIPSA showed significant soft tissue preservation. NIPSA may represent a promising papillae preservation technique in the treatment of intraosseous periodon- tal defects.
Aslan et al., 2017 [31]	“Entire papilla preservation technique in the regenerative treatment of deep intrabony defects: 1-Year results.”	Prospective case series	To report the clinical outcomes and potential benefits of a surgical “tunnel-like” approach in managing deep, isolated intrabony lesions.	Presented technique reduces wound failure risk, particularly in early healing, preventing biomaterial exposure, stabilizing blood clots in deep intrabony defects, and improving clinical outcomes.
Shi et al., 2023 [32]	“A novel periodontal endoscopy-aided non-incisional periodontal regeneration technique in the treatment of intrabony defects: a retrospective cohort study.”	Retrospective cohort study	To explore the feasibility of periodontal endoscopy-aided NIT in comparison with periodontal endoscopy- aided SRP (PSRP).	By eliminating flap elevation, PE-aided NIT preserves an optimal periodontal microenvironment for regeneration, making it a potential alternative technique for treating intrabony defects.

**Table 2:** Tabular presentation of quantitative clinical outcomes.

Author/Year	Defects (n), Diagnostic Criteria for Defects	Follow-Up	Smoking, Mean Age	Type of Procedure	Outcomes	CAL (mm) Mean ± SD	PD (mm) Mean ± SD	REC/GMP (mm) Mean ± SD
Sanz et al., 2024 [21]	6, PD ≥ 6 mm with CAL ≥ 6 mm, ≥3 mm in depth with at least two bony walls	6 months	No, 48 ± 13.07 years	EPPT	Average change in PD, CAL, GMP, PP, PW, KTW	3.67 ± 1.03 (*p* < 0.05)	4.00 ± 0.63 (*p* < 0.05)	0.33 ± 0.52
Kobe et al., 2024 [22]	20, periodontitis stage III/IV, at least one deep isolated 2/3-wall intraosseous defect, (PD) ≥ 5 mm and (CAL) ≥ 6 mm	12 months	4 smokers, 16 non-smokers, 53 ± 9 years	Control: collagenous corticocellular xenogeneic bone graft + EPPT/NIPSA Test: prehydrated collagen-containing corticocellular heterologous bone gel + EPPT/NIPSA	Average change in CAL, REC, PD, and TP in mm.	Control: −3.70 ± 1.83 Test: −3.60 ± 1.51	Control: −3.90 ± 1.66 Test: −3.50 ± 0.97	Control: 0.20 ± 0.79 Test: −0.10 ± 0.99
Górski et al., 2023 [23]	18, Stage III periodontitis, one-wall/two-wall/three-wall, PD ≥ 5 mm, CAL ≥ 6 mm, DD ≥ 3 mm	6 months	No, 42.61 ± 6.94 years	Control: Modified EPPT + EMD + allogeneic bone graft Test: Modified EPPT + EMD + allogeneic bone graft + SCTG	Average change in PD, CAL, REC, KTW, DD, FMPS, FMBS	Control: −4.87 ± 1.36 mm (*p* < 0.0001) Test: −4.66 ± 1.98	Control: −4.33 ± 1.25 mm (*p* < 0.0001) Test: −4.67 ± 2.08	Control: −0.03 ± 0.48 Test: −0.5 ± 0.5
Pohl et al., 2023 [24]	6, Three-wall/two-wall, CAL ≥ 6 mm	Average 30 months	No, 37–54 years	VISTA + CTG + CCTB	PPD, CAL, REC, PT	Initial: 8.5 ± 0.83 Post: 2.7 ± 0.52	Initial: 8.2 ± 0.75 Post: 2.7 ± 0.52	Initial: 0.3 ± 0.52 Post: 0
Moreno Rodríguez et al., 2022 [25]	24, periodontitis stage III and IV, grade A, PD > 6 mm and extension of the intrabony defect > 3 mm, 1 and/or 2 walls	12 months	No, Control: 46.50 ± 10.47 years Test: 50.33 ± 9.02 years	Control: NIPSA + EMD Test: NIPSA + EMD + BS	Average change in BOP, PPD, CAL, REC, PT, KTW, SUPRA-AG	Control: 8.33 ± 2.74 Test: 7.08 ± 2.68	Control: 8.25 ± 2.70 Test: 6.83 ± 0.81	Control: − 0.25 ± 0.45 (increased) Test: − 0.17 ± 0.58 (increased)
Calzavara et al., 2021 [26]	9, periodontitis stage III/IV, 1 or 2 walls, residual PPD ≥ 6 mm and intrabony component ≥ 3 mm	18 months (7 cases) 5 years (2 cases)	No	AICAST + EMD + bovine bone-derived xenograft	Average change in PPD, CAL, REC	18 months follow up cases: 5.66 ± 0.73 5-years follow up cases: 7.42 ± 4.12	18 months follow up cases: 6.81 ± 2.19 5-years follow up cases: 8.58 ± 1.53	18 months follow up cases: 1.14 ± 2.01 5-years follow up cases: 1.16 ± 2.59
Aslan et al., 2021 [27]	15, Periodontitis stage III/IV, 1 or 2 wall, (PD) ≥ 6 mm, (CAL) ≥ 6 mm and at least 4 mm intrabony component in the interdental area	12 months	No, 47.73 ± 12.18	EPPT + depro-teinized bovine-derived bone substitute + collagen barrier	Average change in PPD, CAL, REC	5.86 ± 1.28 (*p* < 0.0001)	6.1 ± 1.47 (*p* < 0.0001)	0.23 ± 0.62 (increased)
Aslan et al., 2020 [28]	30, isolated intrabony defect (PD) ≥ 7 mm, (CAL) ≥ 8 mm and an intrabony component ≥ 4 mm measured radiographically	12 months	No, 43.93 ± 12.85 years	Control: EPPT Test: EPPT + EMD + bovine- derived bone substitutes (BS)	Average change in PPD, CAL, REC	Control: 5.83 ± 1.12 Test: 6.3 ± 2.5	Control: 6.2 ± 1.33 Test: 6.5 ± 2.65	Control: −0.36 ± 0.54 (increased) Test: −0.2 ± 0.25 (increased)
Moreno Rodríguez et al., 2019 [29]	20, (PD) > 5 mm, and an intrabony component ≥ 4 mm	12 months	5 smokers and 7 former smokers, 30–60 years	NIPSA + EMD + deproteinized bovine bone xenograft	Average change in PPD, CAL, REC, TP, KTW, SUPRA-AG	5.9 ± 2.38 (<0.001)	5.6 ± 2.48 (<0.001)	0.25 ± 0.44
Moreno Rodríguez et al., 2019 [30]	30, (PD) > 5 mm, intrabony defect > 3 mm, intrabony defect configuration including a 1 and/or 2-wall involving the buccal wall	12 months	14 smokers, 16 non-smokers, 44.36 ± 5.9 years	Control: MIST + EMD + deproteinized bovine bone xenograft Test: NIPSA + EMD + deproteinized bovine bone xenograft	Average change in PPD, CAL, REC, TP, KTW,	MIST: 3.6 ± 1.40 *p* < 0.001 NIPSA: 5.33 ± 2.47 *p* < 0.001	MIST: 4.33 ± 1.45 *p* < 0.001 NIPSA: 5.53 ± 2.56 *p* < 0.001	MIST: −0.73 ± 0.88 (increased) NIPSA: −0.20 ± 0.41 (increased)
Aslan et al., 2017 [31]	12, isolated two- or three-wall intrabony defect with (PD) ≥ 7 mm, (CAL) ≥ 7 mm and at least 4 mm intrabony component	12 months	No, 42.6 ± 13.1 years	EPPT + EMD + deproteinized porcine-derived bone substitute	Average change in PPD, CAL, REC	6.83 ± 2.51 *p* < 0.001	7 ± 2.8 *p* < 0.001	0.16 ± 0.38
Shi et al., 2023 [32]	117, stage III/IV periodontitis, at least one tooth with probing depth (PD) ≥ 5 mm and bleeding on probing, at least one intrabony defect with the depth ≥ 3 mm	12 months	No, NIT: 21 subjects (32.67 ± 5.83 years) PSRP: 21 subjects (35.76 ± 9.63 years)	Control: periodontal endoscopy- aided SRP (PSRP) Test: periodontal endoscopy-aided non-incisional regeneration technique (NIT)	Average change in PPD, CAL, REC, IBD	PSRP: −2.38 ± 1.60 NIT: −3.62 ± 2.70	PSRP: −3.07 ± 1.66 NIT: −4.14 ± 2.16	PSRP: 0.70 ± 1.15 (decreased) NIT: 0.71 ± 0.90

## Abbreviations

BD	Base of the Defect
CAL	Clinical Attachment Level
CAL-G	Clinical Attachment Level Gain
CEJ	Cementoenamel Junction
DBBM	Deproteinized Bovine Bone Mineral
DD	Radiographic Defect Depth
FMBS	Full-Mouth Bleeding Score
FMPS	Full-Mouth Plaque Score
GTR	Guided Tissue Regeneration
MPPT	Modified Papilla Preservation Technique
n	Number of Defects
OFD	Open-Flap Debridement
PPD	Probing Pocket Depth
PPD-R	Probing Pocket Depth Reduction
RCT	Randomized Controlled Trial
SD	Standard Deviation
PPT	Papilla Preservation Technique
EPPT	Entire Papilla Preservation Technique
MIST	Minimally Invasive Surgical Technique
M-MIST	Modified Minimally Invasive Surgical Technique
NIPSA	Non-Incised Papillae Surgical Approach
SRP	Scaling and Root Planing
NIT	Non-Incisional Regeneration Technique
PESRP	Periodontal Endoscopy-Aided Scaling and Root Planning
SFA	Single-Flap Approach
IBD	Intra-Bony Defect
